# The pseudopeptide HB-19 binds to cell surface nucleolin and inhibits angiogenesis

**DOI:** 10.1186/2045-824X-4-21

**Published:** 2012-12-24

**Authors:** Charalampos Birmpas, Jean Paul Briand, Josẻ Courty, Panagiotis Katsoris

**Affiliations:** 1 Department of Biology, University of Patras, Patras, Greece; 2 CNRS, Université Paris Est Créteil, Créteil Cedex, France; 3 CNRS, Institut de Biologie Moléculaire et Cellulaire, Strasbourg, France

## Abstract

**Background:**

Nucleolin is a protein over-expressed on the surface of tumor and endothelial cells. Recent studies have underlined the involvement of cell surface nucleolin in tumor growth and angiogenesis. This cell surface molecule serves as a receptor for various ligands implicated in pathophysiological processes such as growth factors, cell adhesion molecules like integrins, selectins or laminin-1, lipoproteins and viruses (HIV and coxsackie B). HB-19 is a synthetic multimeric pseudopeptide that binds cell surface expressed nucleolin and inhibits both tumor growth and angiogenesis.

**Methodology/principal findings:**

In the present work, we further investigated the biological actions of pseudopeptide HB-19 on HUVECs. In a previous work, we have shown that HB-19 inhibits the *in vivo* angiogenesis on the chicken embryo CAM assay. We now provide evidence that HB-19 inhibits the *in vitro* adhesion, migration and proliferation of HUVECs without inducing their apoptosis. The above biological actions seem to be regulated by SRC, ERK1/2, AKT and FAK kinases as we found that HB-19 inhibits their activation in HUVECs. Matrix metalloproteinases (MMPs) play crucial roles in tumor growth and angiogenesis, so we investigated the effect of HB-19 on the expression of MMP-2 and we found that HB-19 downregulates MMP-2 in HUVECs. Finally, down regulation of nucleolin using siRNA confirmed the implication of nucleolin in the biological actions of these peptides.

**Conclusions/significance:**

Taken together, these results indicate that HB-19 could constitute an interesting tool for tumor therapy strategy, targeting cell surface nucleolin.

## Introduction

Nucleolin is a nucleolar protein ubiquitously expressed in exponentially growing eukaryotic cells. It was first described in 1973 as a protein involved in ribosome biogenesis and also in DNA and RNA metabolism [1]. More recently, nucleolin was shown to shuttle between cytoplasm and cell surface. In the cytoplasm, it provides a post-transcriptional regulation of mRNA and at the cell surface it serves as a low affinity receptor for several ligands such as growth factors [2,3]. Cell surface nucleolin was first described in hepatocarcinoma cells, suggesting that this molecule is involved in the growth of tumor cells [4].

Since this report, a growing body of evidence has pointed out the involvement of cell-surface expressed nucleolin in cell proliferation, and more specifically in tumor cell growth and angiogenesis. Surface nucleolin expression is constantly enhanced in various tumor cell lines and activated endothelial cells [2,5,6] and this expression is constantly induced in these cells [7]. The expression of nucleolin is enhanced on the surface of endothelial cells upon stimulation with the vascular endothelial growth factor (VEGF) [8]. The functional blockade or downregulation of surface nucleolin in endothelial cells inhibits their migration and prevents capillary-tubule formation [8]. Furthermore, several molecules related to cell proliferation or differentiation, have been reported as ligands for cell surface nucleolin. Among these molecules are the hepatocyte growth factor (HGF), the heparin affin regulatory peptide (HARP), midkine (MK), the epithelial growth factor receptor (ErbB) and endostatin, which all play a significant role in tumor development and angiogenesis processes [9-12]. In addition, molecules involved in mechanisms regulating pericellular proteolysis, cell-surface adhesion and mitogenesis (such as urokinase) bind and are co-internalized with surface nucleolin [13,14]. Other surface nucleolin binding proteins such as laminin-1, factor J, L- and P-selectins are involved in cell differentiation and regulate cell adhesion, leukocyte trafficking, inflammation and angiogenesis [15-19]. Cell surface nucleolin, as a novel target for anticancer therapy, was validated by using several molecules such as endostatin, the aptamer AS1411, the acharan sulfate and the homing tumor peptide F3 [20-22]. Furthermore, targeting nucleolin using a specific antibody causes activated endothelial cells apoptosis by decreasing the anti-apoptotic bcl2 mRNA in tumor vasculature [23].

In a previous study, we have reported that the nucleolin binding multivalent pseudopeptide HB-19 suppressed both tumor growth and angiogenesis [24]. HB-19 binds the RGG domain located in the C-terminal part of nucleolin and leads to its internalization [9,24]. *In vitro*, HB-19 reduces tumor cell growth in soft agar assay in several carcinoma cell lines and impairs endothelial cells proliferation, migration and differentiation induced by VEGF [24]. These activities in both tumor and activated endothelial cells lead to tumor growth inhibition in breast carcinoma MDA-MB 231 and human melanoma MDA-MB 435 xenografts in athymic nude mice, without displaying any toxicity in normal tissues [24].

In this study, we have further investigated the anti-angiogenic activities and the mechanism of action of HB-19 on human umbilical vein endothelial cells (HUVECs).

## Materials and methods

### Ethics statement

For this study, we have obtained ethics approval from the ethics committee of University of Patras.

### Endothelial cell culture

Human umbilical vein endothelial cells (HUVECs) were isolated from the umbilical cord vein by collagenase digestion as previously described [25] and used at passages 2–4. The cells were grown as monolayers in medium M199 supplemented with 15% fetal bovine serum (FBS), 150 μg/ml of endothelial cell growth supplement, 5 U/ml heparin sodium, 100 U/ml penicillin-streptomycin and 50 μg/ml gentamycin. Cultures were maintained at 37°C, 5% CO_2_ and 100% humidity.

### Cell proliferation assay

An equal number of cultured HUVECs in medium containing 15% FBS were left to adhere for 20 h in a cell culture microplate. They were then treated with various HB-19 concentrations and were allowed to proliferate for 3 days. The cell number was estimated by the crystal violet assay. Data are the mean ± SEM of at least three independent experiments.

### Boyden chamber assay

Migration assays were performed as previously described [26] in a 24-well microchemotaxis chamber (Costar, Avon, France), using untreated polycarbonate membranes with 8 μm pores. HUVECs were harvested and resuspended at a concentration of 10^5^ cells/0.1 ml in the corresponding medium containing 0.25% bovine serum albumin (BSA) and HB-19. The bottom chamber was filled with 0.6 ml of the corresponding medium containing 0.25% BSA. The upper chamber was loaded with 10^5^ cells and incubated for 4 h at 37°C. After completion of the incubation, filters were fixed, non-migrated cells were scrapped off the upper side of the filter, and filters were stained with crystal violet. The number of migrated cells was quantified by counting the entire area of the filter using a grid and an Optech microscope at a 20x magnification.

### In vitro endothelial cell wound healing assay

HUVECs were cultured in 6-well plates 2×10^5^ cells/well as confluent monolayers. The monolayers were wounded in a line across the well with a 200 μl standard pipette tip, washed twice with PBS to remove cell debris and incubated with increasing concentrations of HB-19 for 48 h. The area of the initial wound was photographed using a charge-coupled device camera connected to an inverted microscope (Axiovert 35; Zeiss, Thornwood, NY). The wound healing effect was calculated in comparison with the area of the initial wound.

### Western blot analysis

Cells were starved for 4 h and then incubated with various concentrations of HB-19 for 15 min. Cells were subsequently washed twice with PBS and lysed in 250 μl 2× SDS loading buffer under reducing conditions. Proteins were separated by SDS-PAGE and transferred to an Immobilon-P membrane for 3 h in 48 mM Tris pH 8.3, 39 mM glycine, 0.037% SDS, and 20% methanol. The membrane was blocked in TBS containing 5% non-fat milk and 0.1% Tween 20 for 1 h at 37°C. Membranes were incubated with primary antibody overnight at 4°C under continuous agitation, washed with PBS-Tween and incubated with the appropriate secondary antibody coupled to horseradish peroxidase. Protein bands were detected using the ChemiLucent Detection System Kit (Chemicon International Inc., CA) according to the manufacturer’s instructions. Where indicated, blots were stripped in buffer containing 0.5 mM Tris HCl pH 6.8, 2% SDS, 100 mM 2-mercaptoethanol for 30 min at 56°C and re probed. A quantitative estimation of band size and intensity was performed through analysis of digital images using the ImagePC image analysis software (Scion Corporation, Frederick, MD).

### Gelatin zymography

Secreted metalloproteinases were detected and characterized by zymography. Conditioned media were obtained after an 8 h incubation of cells in serum-free media and then were loaded onto 10% SDS-PAGE gels that had been co-polymerized with 1 mg/ml gelatin. Electrophoresis was carried out under non-reducing conditions at 100 V for 2 h at 4°C. Gels were washed once for 60 min in 2.5% Triton X-100 to remove SDS and incubated in zymogen activation buffer (50 mM Tris–HCl pH 7.6, 10 mM CaCl_2_, 0.2M NaCl) for 24 h at 37°C. Gels were stained with 0.5% Coomassie blue in 30% methanol/10% acetic acid for 30 min at room temperature and de-stained in 30% methanol/10% acetic acid three times for 15 min. The presence of metalloproteinases was indicated by an unstained (due to proteolysis) zone in the substrate. Both active forms and pro-enzymes are revealed by this technique, since exposure of pro-MMPs to SDS during SDS-PAGE leads to activation without proteolytic cleavage. The relative amounts of MMPs were quantified by NIH Image Analysis software. The normalization was based on the number of cells of each well (using the crystal violet method).

### Annexin V binding staining

The analysis of annexin V binding was carried out with an Annexin V-FITC Detection Kit I (PharMingen, San Diego, CA) according to the manufacturer’s instructions. An equal number of cultured HUVECs in medium containing 15% FBS were left to adhere for 20 h in a cell culture microplate. They were then treated with various HB-19 concentrations and collected after 24 h, washed twice with cold PBS, centrifuged at 200g for 5 min and resuspended in binding buffer at a concentration of 10^6^ cells per ml. 100 μl of the solution were transferred to a 5ml culture tube and 5 μl of annexin V-FITC and 5 μl of PI were added. Cells were gently vortexed and incubated for 15 min at room temperature in the dark. Finally, 400 μl of binding buffer were added to each tube, and samples were analyzed by FACScan flow cytometer (Becton Dickinson). For each sample, 10,000 ungated events were acquired. PI (−)/annexin (+) cells represent early apoptotic populations and PI (+)/annexin (+) cells represent late apoptotic populations.

### Crystal violet assay

Adherent cells were fixed with methanol and stained with 0.5% crystal violet in 20% methanol for 20 min. After gentle rinsing with water, the retained dye was extracted with 30% acetic acid, and the absorbance was measured at 595 nm.

### Reverse transcriptase-polymerase chain reaction (RT-PCR) for nucleolin, MMP2, and GAPDH

Total RNA was extracted using the Nucleospin RNA II kit (Macherey-Nagel, Germany) according to the manufacturer’s instructions. The integrity of isolated RNA was examined by electrophoresis on a 1% agarose gel containing 0.5 mg/ml ethidium bromide. Specific primers were as follows:

Nucleolin,

5^′′^- TGCCAAGAAGACAGTTACACCA −3^′′^ and

5^′′^- AGGAACAACTTTTGCAGCTTTC - 3^′′^;

MMP2,

5^′′^- ACAGTCCGCCAAATGAACC - 3^′′^ and

5^′′^- CCTGGGCAACAAATATGAGA −3^′′^

GAPDH,

5^′′^-CCACCCATGGCAAATTCCATGGCA-3^′′^ and

5^′′^ TCTAGACGGCAGGTCAGGTCCACC-3^′′^.

The RT-PCR reactions were performed in a single step with 250 ng of total RNA, using the Qiagen RT-PCR system. The RT-PCR products were subjected to electrophoresis on 1% agarose gel containing 0.5 mg/ml ethidium bromide, digitally photographed, and quantified using image analysis software (Scion Image PC, Scion Corporation, Frederick, MD).

### ***SiRNA transfection***

RNA oligonucleotide primers were obtained from Ambion Inc and the Lipofectamine RNAiMAX Transfection Agent was obtained from Invitrogen. The following sequences were used:

siRNA Nucleolin sense : 5^′′^-GGAUAGUUACUGACCGGGA-3^′′;^

siRNA Nucleolin antisense: 5^′′^-UCCCGGUCAGUAACUAUCC-3^′′^,

HUVECs were plated in 6 wells-plates and incubated for 24 hours at 37°C. Cells were then transfected at a final concentration of 10nM siRNA using Lipofectamine RNAiMAX reagent (Invitrogen) according to the manufacturer's instructions.

Transfection efficiency was evaluated using Silencer FAM Labelled GAPDH siRNA (Ambion). Negative control siRNAs from Ambion was also used.

### Materials

Cell culture reagents were from BiochromKG (Seromed, Germany). All other reagents were purchased from Sigma-Aldrich. Monoclonal antibodies against pSRC (Tyr416), pFAK (Tyr925), pAKT (Ser473), pERK1/2 (Thr202/Tyr204), total SRC, total ERK1/2 and were purchased from Cell Signaling Technology. Polyclonal antibody against HSC70 and monoclonal antibody against NCL (nucleolin) were purchased from Santa Cruz Biotechnology, Inc.

### Peptide synthesis

HB-19 was synthesized, as previously described, using the solid phase peptide methodology [8].

### Adhesion assay

24-well culture plates were coated with 1% v/v gelatin for 20 min at 37^0^C. 5×10^4^ resuspended cells incubated in M199 medium with different concentrations of HB-19 for 30 min and then seeded. After a 30 min incubation period, unattached cells were removed by shaking the plates at 2,000 rpm for 10 sec, followed by three washes with PBS. The attached cells number was estimated by the crystal violet assay. Data are the mean ± SEM of at least three independent experiments.

### Statistical analysis

Comparisons of the mean values among groups were performed by means of ANOVA and unpaired Student *t*-test. Homogeneity of variances was tested by Levene’s test. Each experiment included at least triplicate measurements for each condition tested. All results are expressed as mean ± SE. from at least three independent experiments. Values of p less than 0.05 were accepted as significant (*p < 0.05, **p < 0.01, ***p < 0.001).

## Results

### HB-19 inhibits adhesion, proliferation and migration of HUVECs

**Figure 1:**
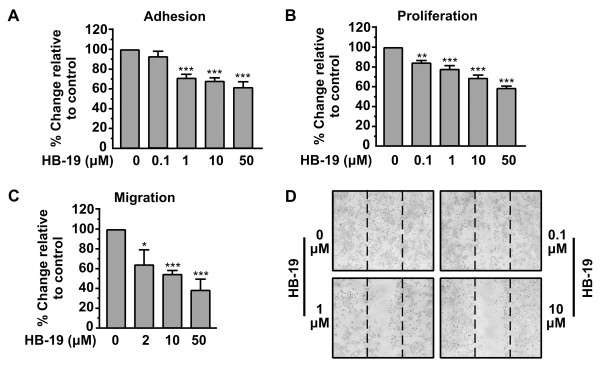
**HB-19 inhibits the *in vitro *adhesion, proliferation, migration and motility of HUVECs.** (**A**) Inhibition of HUVECs adhesion by HB-19. An equal number of HUVECs were incubated with increasing concentrations of HB-19 for 30 min before seeding. After a 45 min incubation period, adherent cells were measured by the crystal violet assay. Results are expressed as % change relative to control and are mean values ± SE from at least 3 independent experiments. (**B**) Inhibition of HUVECs proliferation by HB-19. Cells were cultured for 3 days in presence of increasing concentrations of HB-19. Cell proliferation was quantified by crystal violet staining. Results are expressed as % change relative to control and are mean values ± SE from at least 3 independent experiments. (**C**) Migration of cells through Transwell filters. The lower compartment of Transwell filters (8 μm pores) was filled with growth media containing 0.25% BSA. An equal number of HUVECs was re suspended in growth medium containing 0.25% BSA and increasing concentrations of HB-19, and transferred into Transwell inserts. Cells that successfully migrated through the filter pores, were fixed, stained and quantified by counting the entire area of each filter. Results are expressed as % change relative to control and are mean values ± SE from at least 3 independent experiments. (**D**) Confluent cell monolayers were scratched and cells were left to heal the wound in the presence of increasing concentrations of HB-19. 48 h later the plates were photographed.

The effect of HB-19 on the adhesion of HUVECs was first investigated. As shown in Figure 1A, HB-19 significantly inhibited the *in vitro* adhesion in a concentration-dependent manner, reaching a maximal effect at a concentration of 50 μΜ yielding 40% inhibition compared to the control (Figure 1A). As cells will adhere if left for more than 6 h, we investigated the effect of HB-19 on the proliferation of HUVECs. HB-19 inhibited cell proliferation in a concentration dependent manner, having a maximal effect (41% inhibition relative to control) at a concentration of 50 μΜ (Figure 1B). We then investigated the effect of HB-19 on HUVEC migration, using Transwell assays. Similar to the effects on cell adhesion and proliferation, HB-19 inhibited migration in a concentration-dependent manner, with a maximal effect (61% inhibition relative to control) observed at the concentration of 50 μM (Figure 1C). Furthermore, the effect of HB-19 has been studied in wound-closure assay. As shown in Figure 1D and Additional file 1:, HB-19 inhibits HUVEC motility.

**Figure 2:**
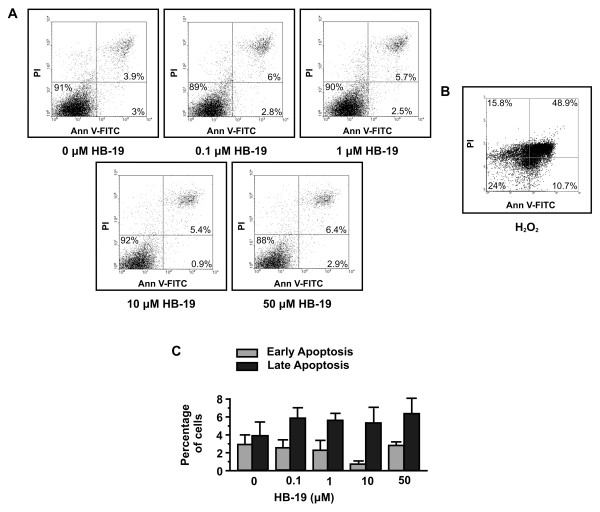
**HB-19 does not induce apoptosis of HUVECs.** (**A** and **C**), endothelial cells were incubated with increasing concentrations of HB-19 and 24 h later the number of apoptotic cells was measured by FACS analysis. (**B**), H_2_O_2_ was used as positive control.

To confirm that HB-19 has a direct effect on adhesion and migration and secondarily on proliferation without affecting cell survival, a cytotoxic assay using HUVEC, treated or not with various concentrations of HB-19, has been performed. As shown in Figure 2, treatment of HUVEC with HB-19 for 24 h did not induce apoptosis (as well as in 48 and 72 h, data not shown). Early apoptotic cells, which are in the beginning of apoptosis, are distinguished from already dead cells (late apoptosis). The data indicated that treatment with HB-19 in various concentrations for 24 h has no effect on the survival of HUVECs and confirmed that HB-19 has a direct effect on endothelial cells adhesion and migration and secondarily on their proliferation.

### HB-19 down-regulates MMP2 in HUVEC

**Figure 3:**
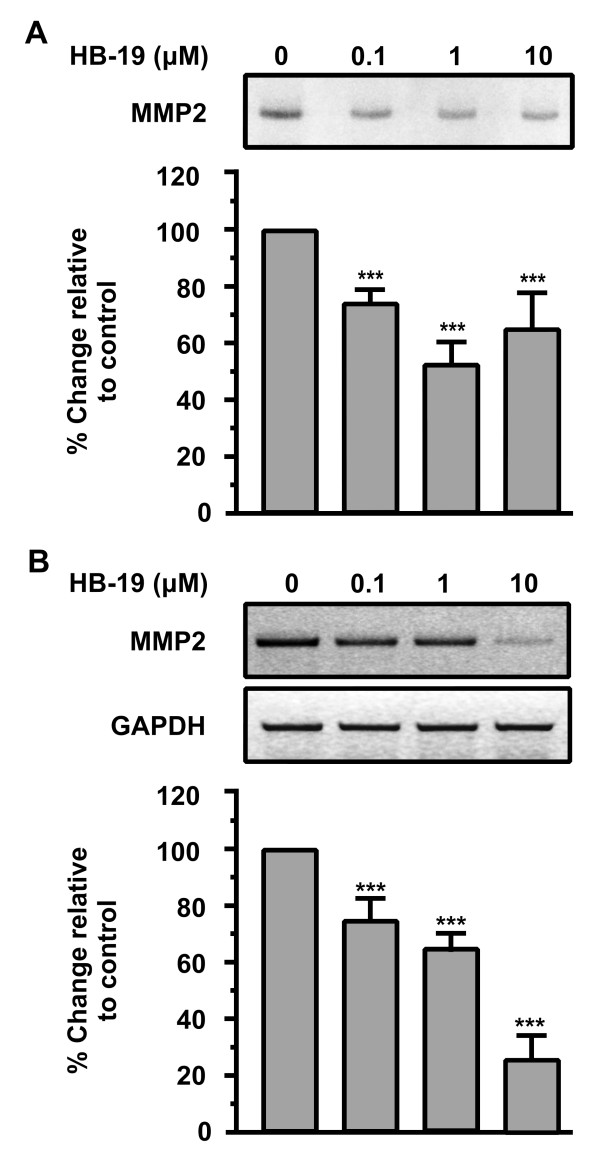
**HB-19 down-regulates MMP2 in HUVECs.** (**A**) Endothelial cells were cultured in a minimal medium with increasing concentrations of HB-19 and 8 h later the supernatants were analyzed for MMP2 activity by zymography. Results are expressed as % change relative to control and are mean values ± SE from at least 3 independent experiments. (**B**) Endothelial cells were incubated with increasing concentrations of HB-19 and 24 h later total RNA was extracted from the cells, RT-PCR reactions were performed using specific primers for MMP2 or GAPDH mRNAs, the PCR products were analyzed in agarose gels and quantified. Results are expressed as % change relative to control and are mean values ± SE from at least 3 independent experiments.

We next studied the effect of HB-19 on the activation of Matrix Metalloproteinases (MMPs) which are involved in the degradation of extracellular matrix, a prerequisite for cell migration. MMPs play a crucial role in angiogenesis, as they digest the ECM and facilitate the cell’s migration. MMP2 is expressed in endothelial as well as in most cell types and is important for endothelial cell migration and vascular remodelling during angiogenesis [27,28]. It also facilitates the migration of tumor cells [29]. Matrix metalloproteinases (MMPs) are crucial in angiogenesis as cells produce them so as to digest ECM and facilitate their migration. Therefore, the effect of HB-19 on the activation of MMPs was investigated. The results showed that MMP2 activity was markedly reduced by 1 μM of HB-19 (Figure 3A). We further demonstrated that HB-19 suppressed the expression of MMP2 mRNA as determined by RT-PCR (Figure 3B). The results indicated that both enzyme activity and the expression of MMP2 were inhibited by HB-19.

The results suggested that HB-19 and therefore nucleolin might affect the expression of genes involved in proteolytic activation.

### Inhibition of SRC, ERK1/2, FAK and AKT activation during HB-19 stimulation of HUVECs *in vitro*

Several studies have indicated that the SRC, FAK, AKT and MAPKs are involved in the signal transduction that regulates angiogenesis [30,31]. Consequently, the effect of HB-19 on the phosphorylation status of SRC, FAK, AKT and ERK1/2 in HUVECs was investigated.

**Figure 4:**
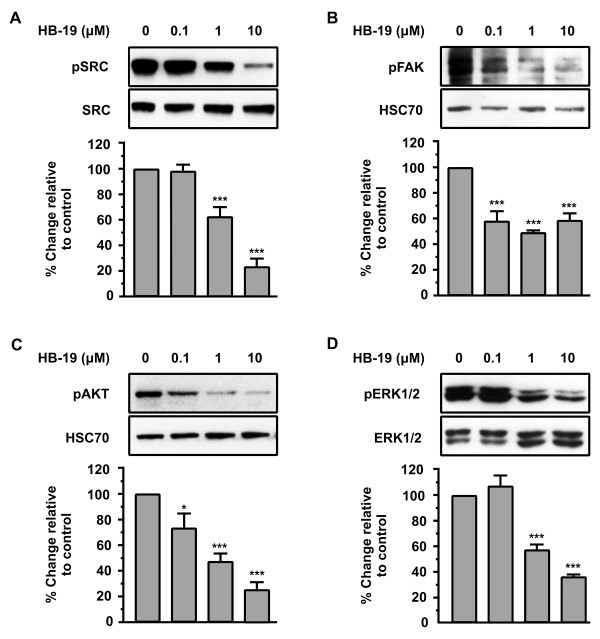
**HB-19 signalling down-regulates pSRC, pFAK, pAKT and pERK1/2.** Western blot analysis of phosphorylated SRC, FAK, AKT and ERK1/2, in cells stimulated by increasing concentrations of HB-19 for 15 minutes. The blots were stripped and re probed for total SRC, HSC70, HSC70 and total ERK1/2 respectively. Results are expressed as % change relative to the control and are mean values ± SE from at least 3 independent experiments.

As shown in Figure 4A, HB-19 induced a decrease in SRC phosphorylation within 15 min and in a concentration dependent manner, with a maximal effect (80% inhibition relative to control) at a concentration of 10 μΜ. We found that FAK phosphorylation was decreased 15 min after incubation of HUVECs with HB-19 in a concentration dependent manner. A significant decrease is observed at a concentration of 1 μM yielding 51% inhibition as compared to the control. Similarly, we found that AKT phosphorylation was decreased 15 min after incubation of HUVECs with HB-19 in a concentration dependent manner with a maximal effect (75% inhibition relative to control) at a concentration of 10 μΜ. As shown in Figure 4D, ERK1/2 were also inactivated after a15 min incubation with HB-19 in a concentration dependent manner, reaching a maximal effect (66% inhibition relative to control) at a concentration of 10μΜ.

Taken together, these data indicate that HB-19 inhibits the phosphorylation of all of these kinases in a dose-dependent manner (Figure 4A, B, C and D). Results showed that the anti-angiogenic action of HB-19 might partly occur through a suppression of SRC, FAK, AKT and ERK pathways, but further research is needed in order to understand the mechanism of action of HB-19 and how it inhibits the activation of these signalling pathways.

### Down regulation of nucleolin expression inhibits adhesion and proliferation of HUVECs and blocks the inhibitory action of HB-19

In order to confirm that HB-19 exerts the previously described biological actions mainly through nucleolin, as well as the involvement of cell-surface nucleolin in these effects, we transiently transfected HUVECs with a siRNA targeting the mRNA of nucleolin. In parallel, HUVECs were transiently transfected with a siRNA that does not target any mRNA (negative control) (data not shown).

**Figure 5:**
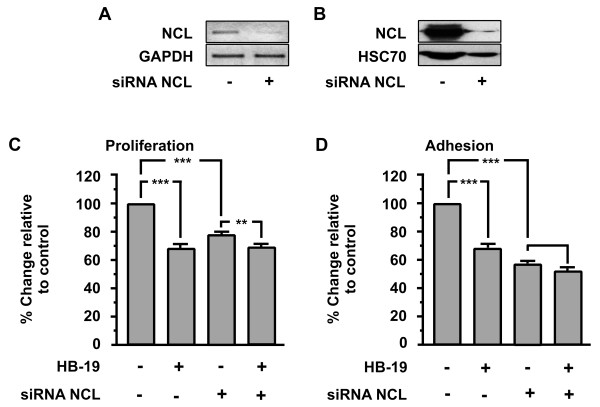
**Effect of nucleolin (NCL) knockdown on HB-19 biological actions.** Down-regulation of nucleolin (NCL) mRNA (**A**) and protein (**B**) using specific siRNA targeting nucleolin mRNA. Effect of HB-19 (10μΜ) on proliferation (**C**) and adhesion (**D**) of HUVECs. The last two bars of each diagram indicate HUVECs that were transiently transfected with siRNA targeting nucleolin (NCL). Results are expressed as % change relative to control and are mean values ± SE from at least 3 independent experiments.

As shown in (Figure 5A and B), using specific siRNA targeting nucleolin mRNA reduces the levels of mRNA and protein up to 80% respectively.

As shown in (Figure 5C and D), NCL knockdown blocked the inhibitory effect of HB-19 in HUVECs proliferation and adhesion respectively. Moreover, the same experiment shows that nucleolin is a crucial factor in the proliferation and adhesion of HUVECs, since the down-regulation of nucleolin expression by itself results in the inhibition of proliferation and adhesion.

### Down regulation of nucleolin expression inhibits SRC and ERK1/2 activation in HUVECs and blocks the inhibitory action of HB-19

Previous reports have shown that HB-19 interacts specifically with cell surface nucleolin [32].

**Figure 6:**
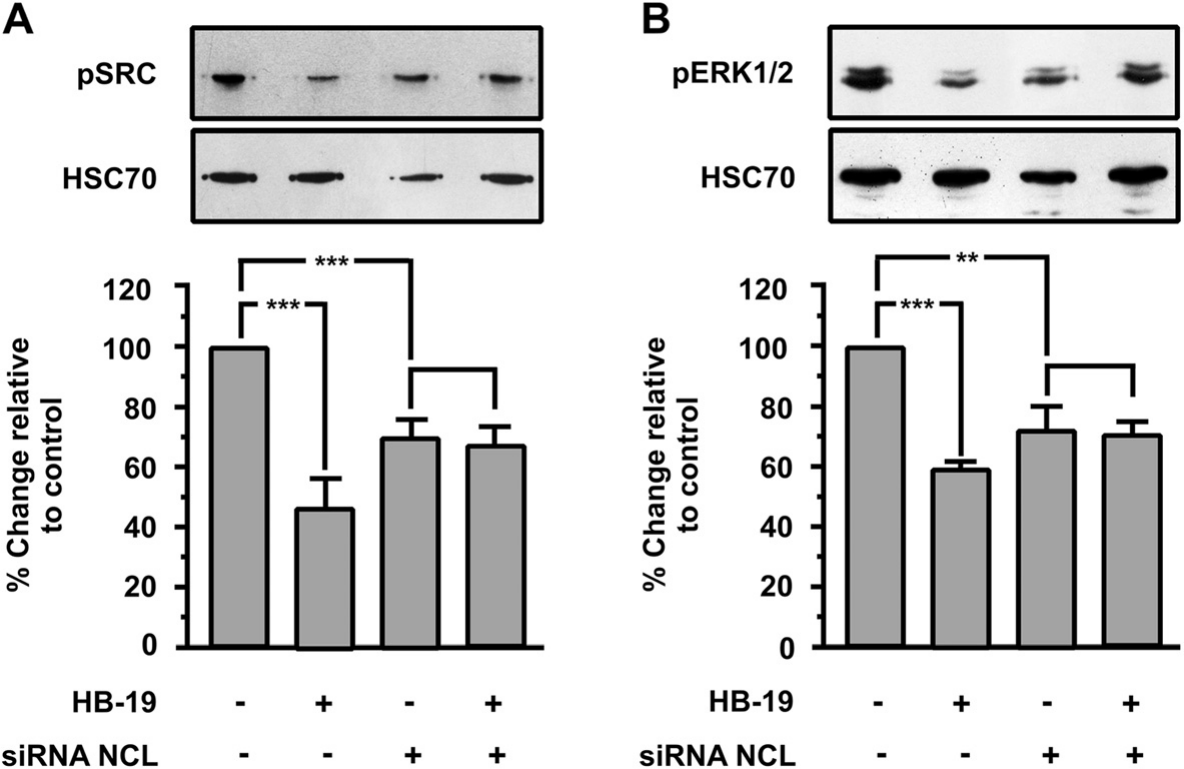
**Effect of nucleolin (NCL) knockdown on HB-19-induced signal transduction.** Western blot analysis of phosphorylated SRC and ERK1/2 in HUVECs stimulated with 10 μM HB-19 for 15 minutes. The blots were stripped and re probed for HSC70. The last two bars of each diagram indicate HUVECs that were transiently transfected with siRNA targeting nucleolin. Results are expressed as % change relative to control and are mean values ± SE from at least 3 independent experiments.

To confirm that HB-19 signalling takes place mainly through nucleolin interaction, we tested the HB-19 effect on activation of SRC and ERK1/2 of transiently transfected HUVECs with siRNA for nucleolin mRNA. As shown in Figure 6A and B, the phosphorylation levels of SRC and ERK1/2 are induced on transiently transfected HUVECs compared with wild type cells, while nucleolin knockdown blocked HB-19-induced SRC and ERK1/2 inactivation.

Taken together, these results indicate that ΗΒ-19/nucleolin interaction affects various signal transduction pathways that reduce the phosphorylation levels of these signal transduction molecules, and are involved in the inhibition of cellular adhesion and proliferation.

## Discussion

Several recent studies have described cell-surface nucleolin as a molecule involved in tumor growth and angiogenesis [2,24,33]. Targeting surface nucleoproteins like nucleolin with HB-19, is effective regarding the inhibition of tumour cell proliferation and impairment of angiogenesis [24,33]. Therefore, functional blockade of surface nucleoproteins seems to result in an inhibition that is not due to a single growth factor implicated in carcinogenesis. Previous studies of our group have shown the dual action of HB-19 on tumour and endothelial cells, and have pointed out surface nucleoproteins as an important anticancer target [24]. The presence of nucleolin on the cell surface is the consequence of active translocation of cytoplasmic nucleolin to the surface upon stimulation of cell proliferation. Surface and cytoplasmic nucleolin are characterized by similar isoelectric points with pI values at about 4.5, whereas nuclear nucleolin is composed of several subspecies with pI values between 4 and 6 [3,32,34]. These observations lead to the suggestion that the expression of surface nucleolin should be differentially regulated compared to its nuclear counterpart [7]. In support to this assumption, it was shown that surface nucleolin is N-glycosylated and that this N-glycosylation is required for the expression and function of nucleolin at the cell surface [35].

HB-19 binds the RGG domain at the C-terminal end of nucleolin, which is also the site for binding of RNA [36,37], rDNA [38], a subset of ribosomal proteins [39], the urokinase-type plasminogen activator [14] and several growth factors [9-12]. The irreversible binding of HB-19 to this RGG domain could then prevent the proper functioning of surface nucleolin, thereby exerting its antagonistic action. In view of the implications of this in tumor growth and angiogenesis, as well as its capability to bind pathogens and diverse range of ligands including low density lipoproteins [1,2,5], it is plausible to suggest that surface nucleoproteins could function as scavenger receptors [40]. Nucleolin was also shown to exist in a 500-kDa protein complex including several other proteins that are implicated in cell signaling, tumor cell adhesion, and other biological actions related to tumorigenesis and angiogenesis. Targeting surface nucleolin with HB-19 could change the organization of this complex leading to antitumoral and antiangiogenic effects. [33]

In this study, we sought to investigate the biological actions of HB-19 in endothelial HUVECs. Our results showed that HB-19 inhibits *in vitro* HUVECs adhesion, migration and motility in a concentration dependent manner (Figure 1A, C and D). If left for more than 6 h, HUVECs will adhere and migrate even in the presence of HB-19 (data not shown), therefore we investigated the effect of HB-19 in the proliferation of HUVECs showing that it is also inhibited, even if it is a secondary effect (Figure 1B). The inhibitory effect of HB-19 on angiogenesis [24] as well as on HUVECs migration and motility may be associated with down regulation of MMP2 [41,42]. We found that both enzyme activity and expression of MMP2 were inhibited by HB-19 (Figure 3). Furthermore, we found that HB-19 treatment shows no toxicity in HUVECs *in vitro*, as HB-19 didn’t induce apoptosis (Figure 2). HB-19 treatment is not toxic in various experimental models *in vitro* as well as *in vivo*. The lack of translocation of HB-19 to the nucleus probably accounts for its lack of toxicity [24,33].

As mentioned above, several molecules that are involved in tumor development and angiogenesis have been reported to be ligands for cell-surface nucleolin. HB-19 binds the C-terminal RGG domain of cell-surface expressed nucleolin and blocks its function as a receptor or binding molecule for various ligands [9,32,43,44]. Additionally, the binding of an extracellular ligand to surface nucleolin has been reported to be involved in the activation of signaling pathways by promoting Ca2+ entry into cells [45].

In order to identify the signalling pathways that HB-19 affects and through which exerts its biological action, we investigated the effect of this peptide on well known angiogenic signalling pathways mediated by many of these ligands of nucleolin.

We found that HB-19 inhibits the phosphorylation levels of SRC, FAK, AKT, and ERK1/2 in a concentration-dependent manner showing that the anti-angiogenic action of HB-19 might partly occur through suppressing SRC, FAK, AKT and ERK pathways.

Down regulating nucleolin, we found that the proliferation and adhesion of HUVECs was inhibited, highlighting the importance of nucleolin in these biological actions (Figure 5C and D). We studied the total effect of nucleolin without distinguishing between surface and nuclear nucleolin but we need to point out that the nuclear nucleolin has longer half life compared to the surface one. To confirm that our siRNA was specific for NCL, we examined the effect of siRNA on the expression of GAPDH mRNA as shown in Figure 5A and other molecules such as tubulin (data not shown). The first comprehensive review of nucleolin was published more than 10 years ago and focused on the problem of ribosomal RNA transcription, maturation, and assembly [46], principally because the expression of this major nucleolar phosphoprotein was directly correlated with ribosomal DNA (rDNA) transcription [46-50]. The focus of nucleolin research has since widened to include chromatin decondensation [51], cytoplasmic nucleolar transport of ribosomal components and preribosomal particles [47], and nucleogenesis [50]. In particular, nucleolin has been shown to be a component of B cell-specific transcription factor [52,53], an autoantigen [54,55], a DNA/RNA helicase [56], DNA-dependent ATPase [57], and a transcriptional repressor [58]. The protein therefore appears to be involved in fundamental aspects of transcriptional regulation, cell proliferation, and growth. Furthermore, nucleolin is required for a correct mitosis, controlled centrosome duplication [59] and plays a crucial role in the cell cycle [60].

The inhibitory effect of HB-19 on cellular proliferation and adhesion was almost completely blocked in the transfected HUVECs compared to the wild type (Figure 5C and D last two columns). Furthermore, the phosphorylation levels of SRC and ERK1/2 are induced on transiently transfected HUVECs compared with wild type cells (Figure 6, first and third columns), while nucleolin knockdown blocked HB-19-induced SRC and ERK1/2 inactivation (Figure 6 last two columns). In Figure 5B and 6 the reduction of HSC70 levels in siRNA transfected cells does not indicate secondary inhibitory effects of our siRNA on the expression of cellular proteins. We used HSC70 for western blot normalization and its reduced levels are a consequence of the reduced number of cells due to the inhibitory effect of NCL knock-down on cell proliferation. Taken together, these results indicate that the complex ΗΒ-19/nucleolin interaction triggers a signal transduction pathway that reduces the phosphorylation levels of these signal transduction molecules, and also that it inhibits cellular adhesion and proliferation. Down regulation of mRNA of NCL using siRNA is reported to reduce surface nucleolin [8]. Additionally, the expression of nucleolin on the surface of endothelial cells is due to the constant induction of nucleolin mRNA, as NCL mRNA and cell-surface-NCL have half-life time of about 45’-90’ whereas nuclear NCL has more than 24h [7]. Therefore, although we did not distinguish between surface and nuclear nucleolin, we can presume with relative safety that cell surface NCL levels were reduced in our experimental conditions. Consequently, we can hypothesize the involvement of surface NCL in HB-19 action and consider NCL to be the main molecule through which HB-19 exerts its action, knowing that there is more research to be done in order to extrapolate that the mechanism by which HB-19 exerts its action is through surface NCL.

In summary, in this study we shed light on the role of cell surface nucleoproteins in the regulation of their binding proteins signalling and activity in HUVECs. These results indicate that HB-19 and other pseudopeptides like NUCANT 6L could constitute an interesting tool for inhibiting angiogenesis. This possibility is reinforced by the fact that these multimeric pseudopeptides are synthetic molecules which lack in tissular toxicity and whose production can be easily upscaled and stable in serum, thus providing novel therapeutic opportunities in proliferative diseases.

## Competing interests

The authors declare that they have no competing interests.

## Authors’ contributions

All authors participated in the design of the study. BC performed all experiments, performed the statistical analysis and drafted the manuscript. BJP constructed and provided the peptides. CJ helped to draft the manuscript. KP conceived of the study, participated in its design and coordination and helped to draft the manuscript. All authors read and approved the final manuscript.

## Supplementary Material

Additional file 1: **HB-19 inhibits *in vitro *wound healing of HUVECs.** The scratched areas were quantified in three random fields in each treatment, and data were calculated from three independent experiments. Results are expressed as % change relative to control and are mean values ± SE from at least 3 independent experiments. (TIFF 57 kb) Click here for file
