# Supplementary figures and images for: The pseudopeptide HB-19 binds to cell surface nucleolin and inhibits angiogenesis
Source: Vasc Cell. 2012 Dec 24;4:21. doi: 10.1186/2045-824X-4-21 (PMC3606460; doi:10.1186/2045-824X-4-21)

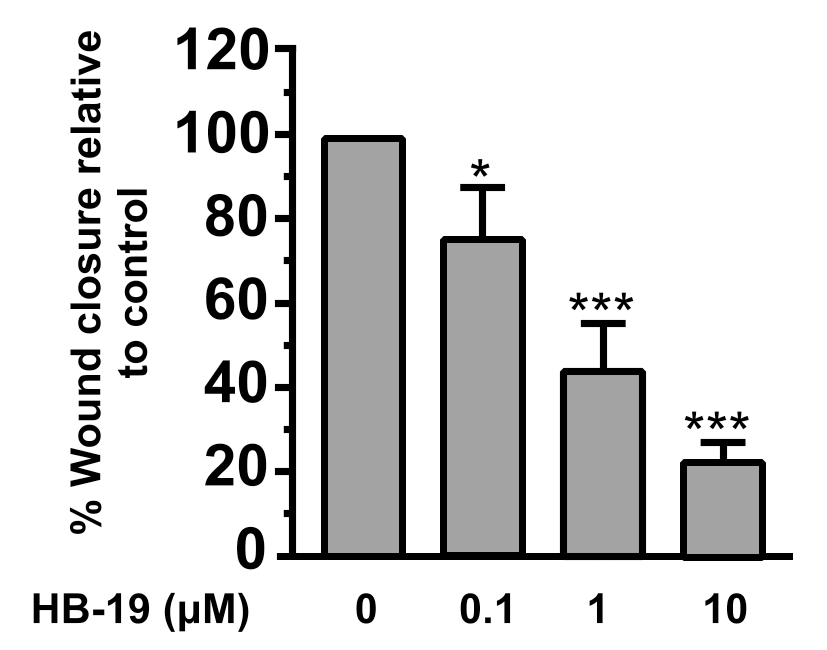

Supplement: Additional file 1: — HB-19 inhibits in vitro wound healing of HUVECs. The scratched areas were quantified in three random fields in each treatment, and data were calculated from three independent experiments. Results are expressed as % change relative to control and are mean values ± SE from at least 3 independent experiments. (TIFF 57 kb) [file 2045-824X-4-21-S1.tiff]
